# Supplementary material for: Fine-tuning mechanical constraints reveals uncoupled patterning and gene expression programs in murine gastruloids
Source: Development. 2025 Sep 29;152(18):dev204711. doi: 10.1242/dev.204711 (PMC12516319; doi:10.1242/dev.204711)
Supplement: Supplementary information [file develop-152-204711-s1.pdf]

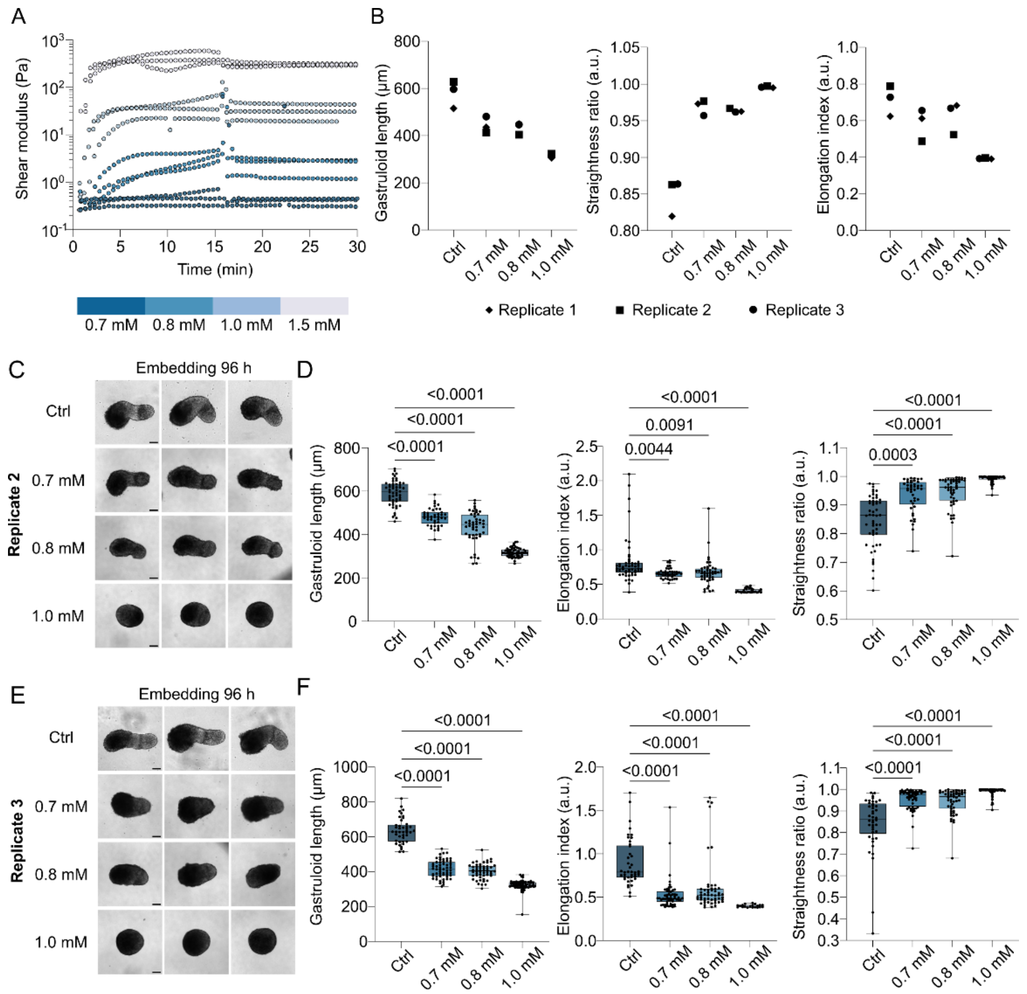

Fig. S1. Effect of gel concentration on gastruloid morphology. (A) Gelation dynamics of hydrogels prepared with different concentrations of components (concentration of reactive functions) (B) Quantification of gastruloid medial axis length, straightness ratio and elongation index as in Fig 1 E-G for Replicates 1, 2 and 3 described in Fig1 and Fig S1 C-F. Median values are represented for each replicate. (C) Replicate 2: Bright field images of gastruloids 120 h after seeding, either grown in 96-well plates or embedded in hydrogel at 96 h after seeding. Gel concentrations 0.7 mM, 0.8 mM or 1.0 mM. Scale bar 100 µm. (D) Replicate 2: Length, elongation index and straightness of gastruloids 120 h after seeding, for gastruloids either grown in 96-well plates or embedded in hydrogel at 96 h after seeding. Gel concentrations 0.7 mM, 0.8 mM or 1.0 mM. Data obtained from bright field images as represented in A: Ctrl N=52; 0.7 mM N=42; 0.8 mM N=48; 1.0 mM N=59. (E) Replicate 3: Bright field images of gastruloids 120 h after seeding, either grown in 96-well plates or embedded in hydrogel at 96 h after seeding. Gel concentrations 0.7 mM, 0.8 mM or 1.0 mM. Scale bar 100 µm. (F) Replicate 3:Length, elongation index and straightness of gastruloids 120 h after seeding, for gastruloids either grown in 96-well plates or embedded in hydrogel at 96 h after seeding. Gel concentrations 0.7 mM, 0.8 mM or 1.0 mM. Data obtained from bright field images as represented in C: Ctrl N=42; 0.7 mM N=56; 0.8 mM N=52; 1.0 mM N=68. Statistical tests were performed using the Kruskal-Wallis test with Dunn’s multiple comparison test.

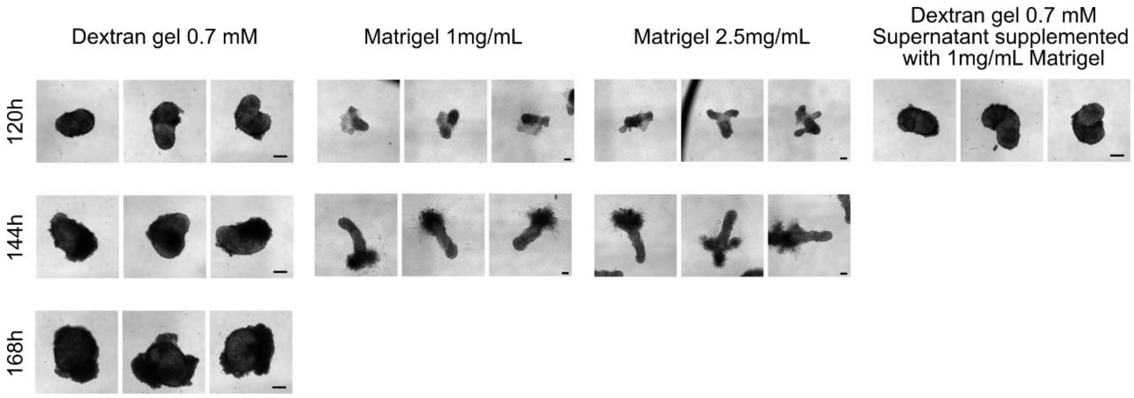

Fig. S2. Differential effects of Matrigel versus bioinert, dextran-based hydrogel and extended embedding. Bright field images of gastruloids 120 h, 144 h or 168 h after seeding, embedding in hydrogel at 96 h after seeding. Hydrogels were Dextran gel at 0.7 mM, Matrigel 1mg/mL, Matrigel 2.5 mg/mL or Dextran gel 0.7 mM where the supernatant was supplemented with 1 mg/mL Matrigel. Scale bar 50 µm

Development • Supplementary information

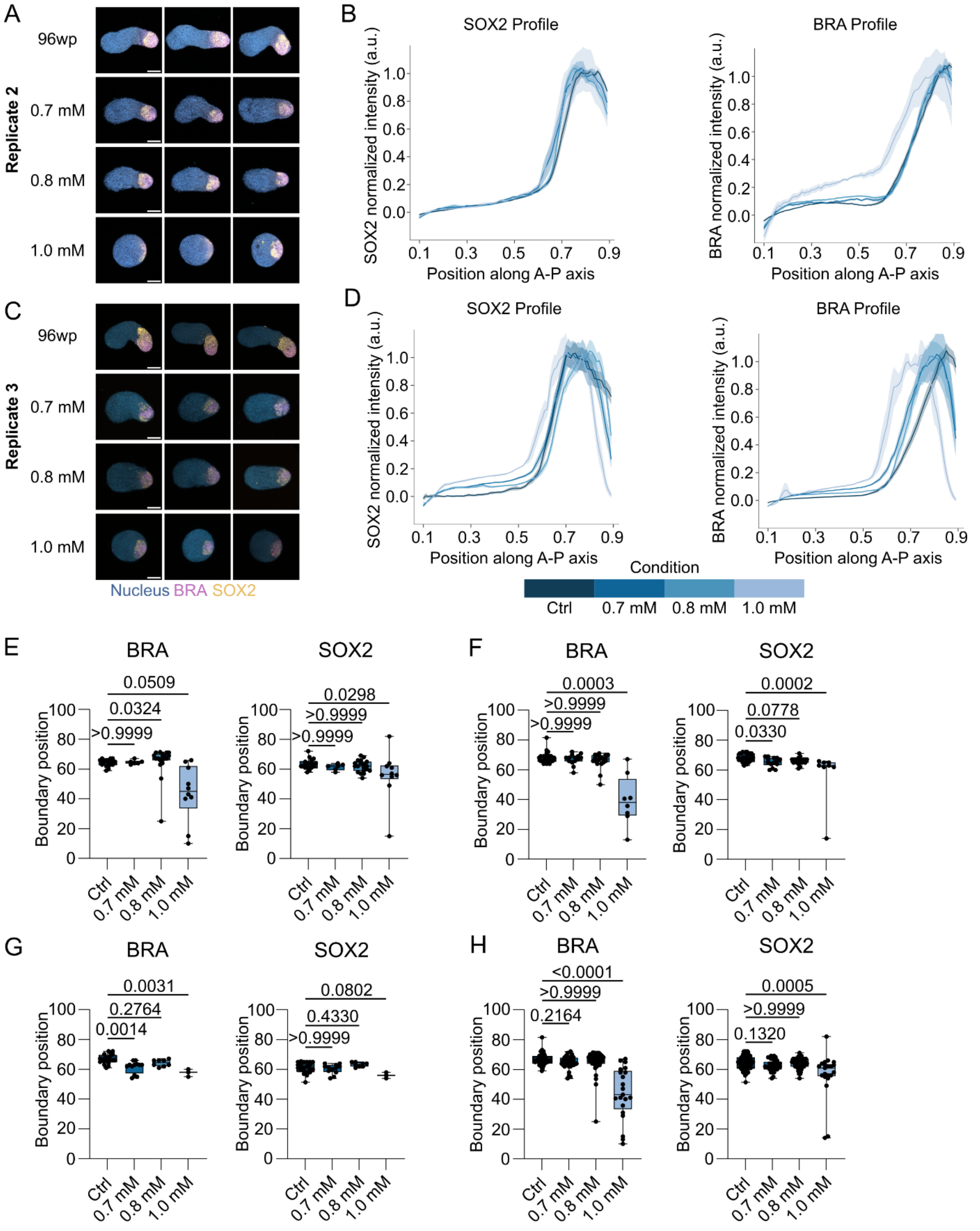

Fig. S3. Posterior gene expression patterning in hydrogel-grown gastruloids. (A) Replicate 2: Immunofluorescence images of gastruloids 120 h after seeding, either grown in 96-well plates or embedded in hydrogel at 96 h after seeding. Gel concentrations 0.7 mM, 0.8 mM or 1.0 mM. Blue: Nucleus, Purple: BRA, Orange: SOX2. Scale bar 100 µm. (B) Replicate 2: Normalized expression profiles (Mean ± SEM) of SOX2 and BRA along the AP axis. Ctrl N=26; 0.7 mM N=14; 0.8 mM N=20; 1.0 mM N=8. (C) Replicate 3: Immunofluorescence images of gastruloids 120 h after seeding, either grown in 96-well plates or embedded in hydrogel at 96 h after seeding. Gel concentrations 0.7 mM, 0.8 mM or 1.0 mM. Blue: Nucleus, Purple: BRA, Orange: SOX2. Scale bar 100 µm. (D) Replicate 3: Normalized expression profiles (Mean ± SEM) of SOX2 and BRA along the AP axis. Ctrl N=21; 0.7 mM N=12; 0.8 mM N=8; 1.0 mM N=3.(E), (F), (G) Boundary positions for BRA and SOX2 profiles for Replicate 1, 2 and 3 respectively from data shown in Figure 2A-C and Figure S2A-D. (H) Pooled values for boundary positions of BRA and SOX2 profiles from Figure S2 E-G. Statistical tests were performed using the Kruskal-Wallis test with Dunn's multiple comparison test.

Development • Supplementary information

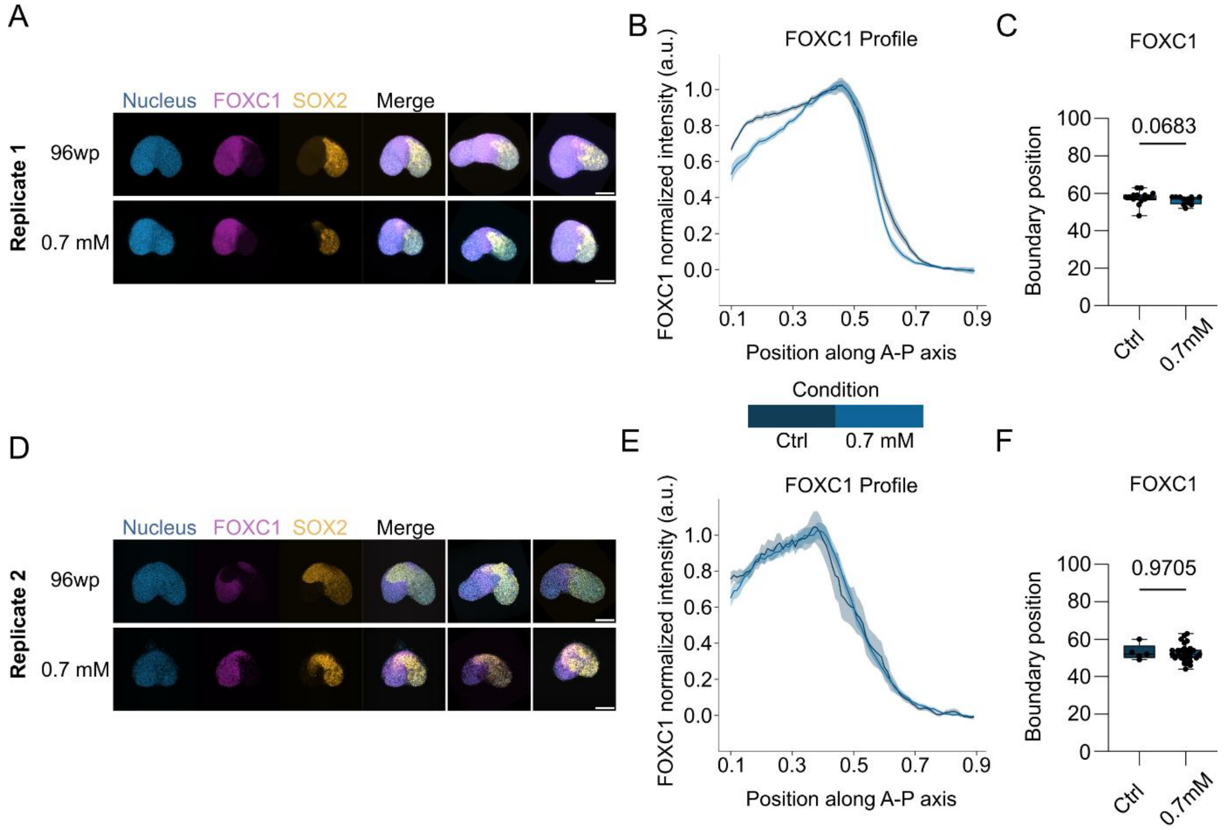

Fig. S4. Anterior gene expression patterning in hydrogel-grown gastruloids. (A) Replicate 1: Immunofluorescence images of gastruloids 120 h after seeding, either grown in 96-well plates or embedded in 0.7 mM hydrogel at 96 h after seeding. Blue: Nucleus, Purple: FOXC1, Orange: SOX2. Scale bar 100 µm. (B) Replicate 1: Normalized expression profiles (Mean ± SEM) of FOXC1 along the AP axis. Ctrl N=14; 0.7 mM N=12. (C) Boundary position for FOXC1 from Replicate 1, from data shown in Figure S3B. (D) Replicate 2: Immunofluorescence images of gastruloids 120 h after seeding, either grown in 96-well plates or embedded in 0.7 mM hydrogel at 96 h after seeding. Blue: Nucleus, Purple: FOXC1, Orange: SOX2. Scale bar 100 µm. (E) Replicate 2: Normalized expression profiles (Mean ± SEM) of FOXC1 along the AP axis. Ctrl N=6; 0.7 mM N=30. (F) Boundary position for FOXC1 from Replicate 2, from data shown in Figure S3E. Statistical test: Mann-Whitney.

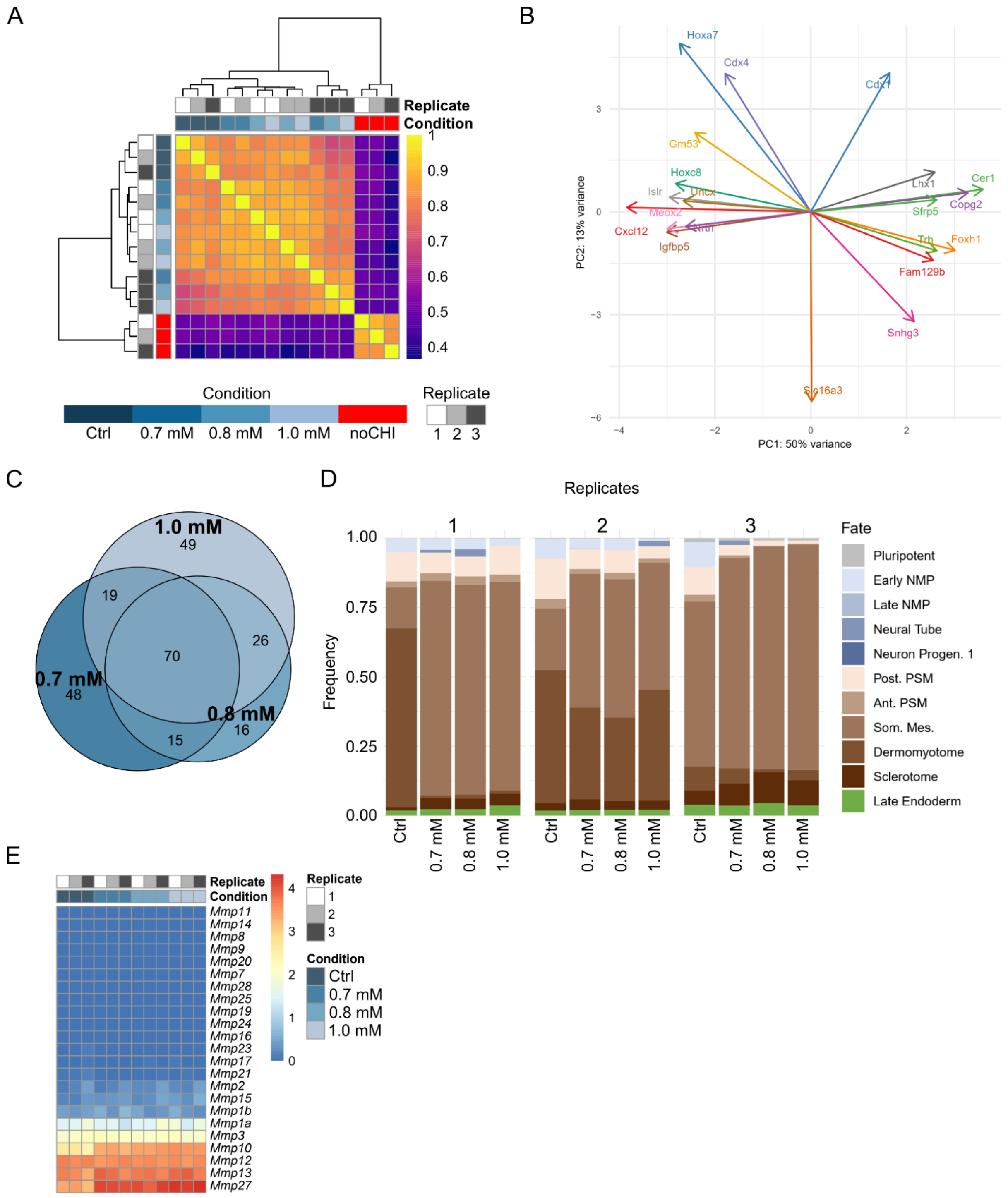

Fig. S5. Detailed RNA-seq analysis of embedded gastruloids. (A) Correlation matrix of bulk RNA sequencing experiments clustered using the ward-D2 algorithm, where noCHI is used as a negative control for gastruloid formation. Colors indicate the Pearson's correlation coefficient values. All samples are analyzed in three independent replicates.(B) Loadings from PCA analysis shown in Figure 2D (C) Euler diagram showing the overlap between DE genes using non-embedded as the control condition. (D) Deconvolution of bulk RNA sequencing. (E) Expression levels of metalloproteases (MMPs) from bulk RNA sequencing.

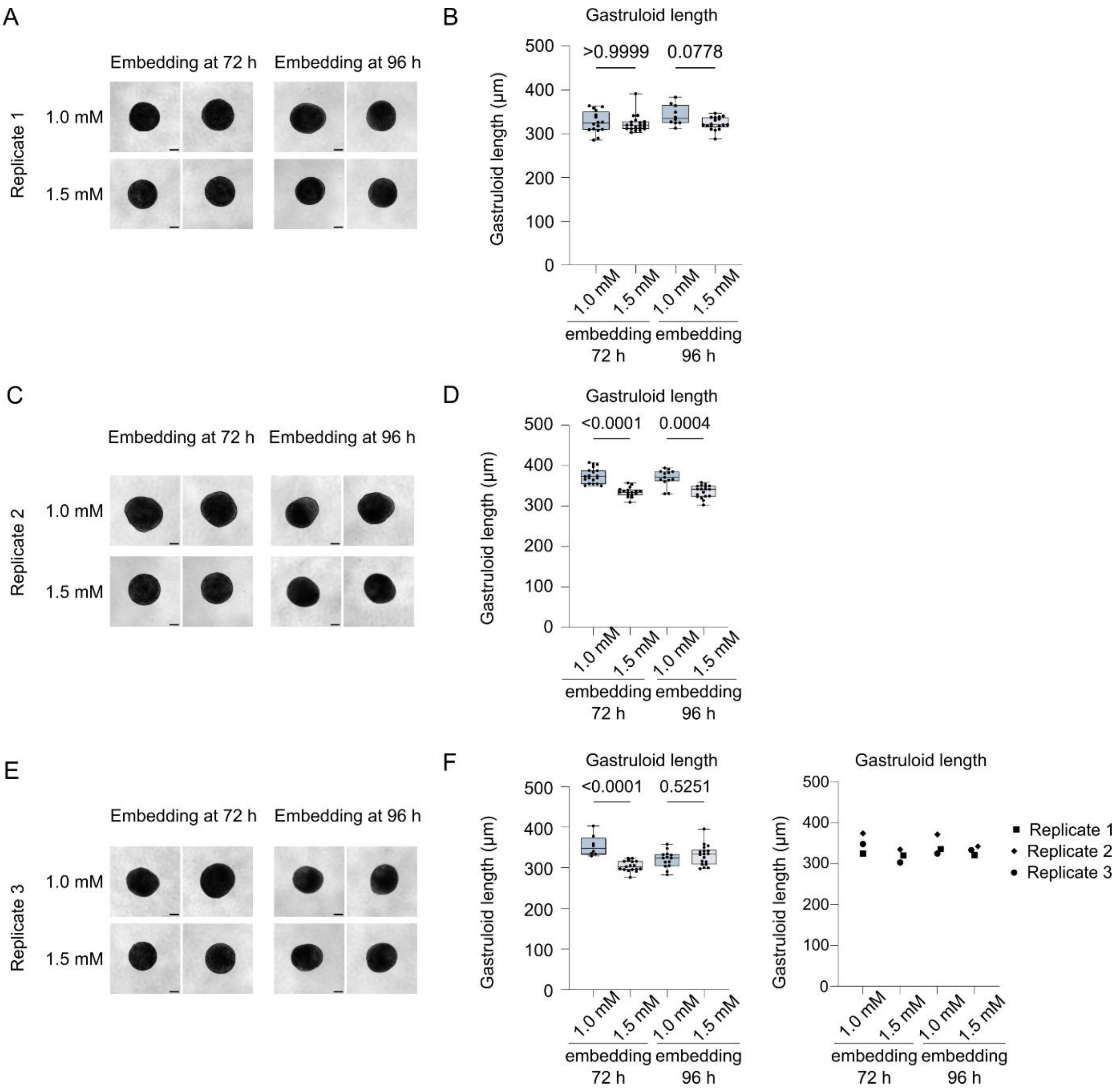

Fig. S6. Effect of high stiffness and early embedding on gastruloid morphology. (A) Replicate 1: Bright field images of gastruloids 120 h after seeding, embedded in hydrogel at 72 h or 96 h after seeding. Gel concentrations 1.0 mM or 1.5 mM. Scale bar 100 µm. (B) Length of gastruloids 120 h after seeding, for gastruloids embedded in hydrogel at 72 h or 96 h after seeding. Gel concentrations 1.0 mM or 1.5 mM. Data obtained from bright field images as represented in A, a representative experiment. Embedded 72 h: 1.0 mM N=16; 1.5 mM N=20. Embedded 96 h: 1.0 mM N=10; 1.5 mM N=18. (C) Replicate 2: Bright field images of gastruloids 120 h after seeding, embedded in hydrogel at 72 h/96 h after seeding. Scale bar 100 µm. (D) Replicate 2: Length of gastruloids 120 h after seeding, for gastruloids embedded in hydrogel at 72 h/96 h after seeding. Data obtained from bright field images as represented in C. 72 h 1.0 mM N=15; 72 h 1.5 mM N=16; 96 h 1.0 mM N=13; 96 h 1.5 mM N=16. (E) Replicate 3: Bright field images of gastruloids 120 h after seeding, embedded in hydrogel at 72 h/96 h after seeding. Scale bar 100 µm. (F) Replicate 3: Length of gastruloids 120 h after seeding, for gastruloids embedded in hydrogel at 72 h/96 h after seeding. Data obtained from bright field images as represented in E. 72 h 1.0 mM N=8; 72 h 1.5 mM N=17; 96 h 1.0 mM N=14; 96 h 1.5 mM N=18. (G) Median value for each replicate of quantification of gastruloid length shown in Fig S3B, D, F. Statistical tests were performed using the Kruskal-Wallis test with Dunn's multiple comparison test. Data shown here correspond to one representative experiment. Experiments were all performed in 3 independent replicates.

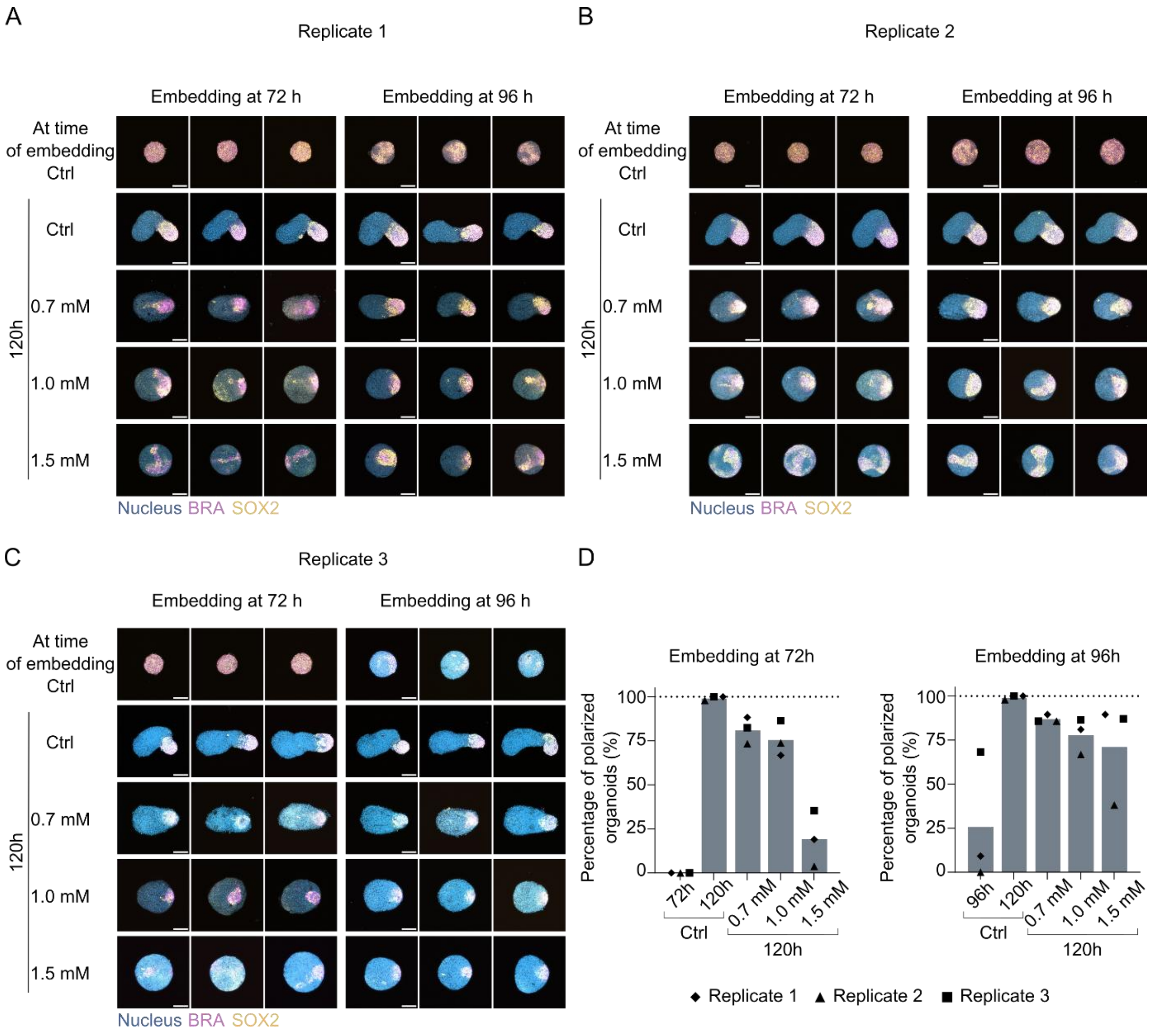

Fig. S7. Effect of embedding timing and gel stiffness on patterning and polarization. (A),(B),(C) For Replicates 1, 2 and 3 respectively: Immunofluorescence images of gastruloids 120 h after seeding, either grown in 96-well plates (Ctrl) or embedded in hydrogel at 72 h/96 h after seeding. Blue: Nucleus, Magenta: BRA, Yellow: SOX2. Scale bar 100 µm. (D) Quantification of the percentage of gastruloids that formed a unique BRA/SOX2 pole at 120 h or at time of embedding, analyzed from immunofluorescence images as in (A), (B) and (C), for gastruloids embedded at 72 h or 96 h. Number of analyzed gastruloids (per replicate): (Left) 72 h N=20/18/22 ; 120h Ctrl N=25/48/24; 0.7 mM N=17/15/17 ; 1.0 mM N=15/19/22 ; 1.5 mM N=21/27/17.(Right) 96 h N=22/23/22 ; 120h Ctrl N= 25/48/24 ; 0.7 mM N=19/21/21 ; 1.0 mM N=21/21/22 ; 1.5 mM N=19/21/23.

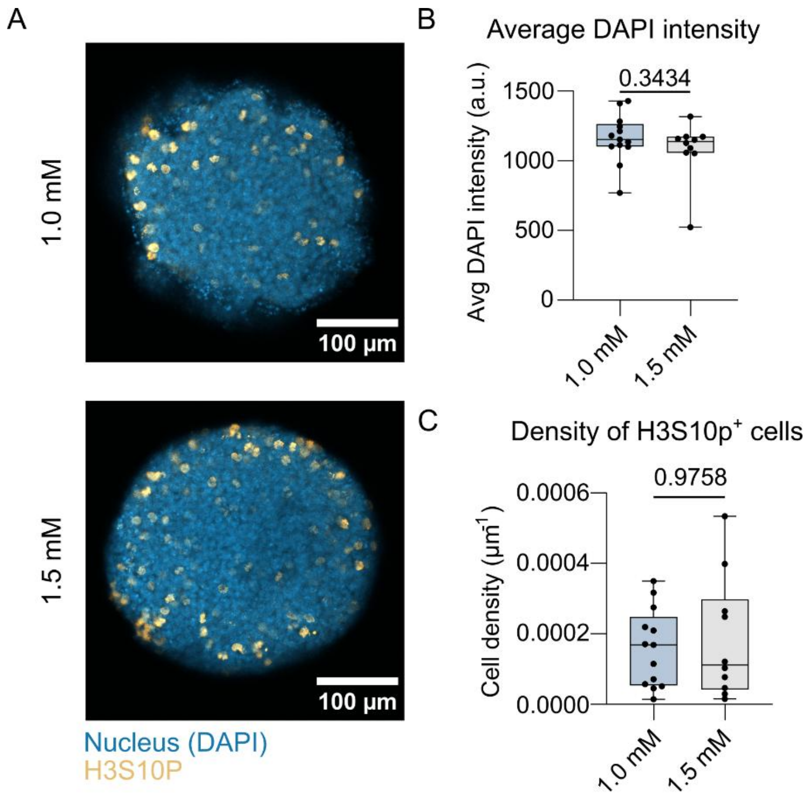

Fig. S8. Cell density and cell proliferation in high stiffness gels. (A) Immunofluorescence images (1 confocal slice) of gastruloids embedded in 1.0 mM or 1.5 mM gels at 72 h, and fixed at 120 h post seeding. Blue: Nucleus. Orange: H3S10P. (B) Average DAPI (nucleus) intensity in one confocal slice and (C) Density of H3S10P high cells in a given depth within the gastruloid (≈80 µm deep). Plotted are Median ± Min/Max. Experiment was performed in 1 replicate, 1.0 mM N=13, 1.5 mM N=10. Statistics: Mann-Whitney test.

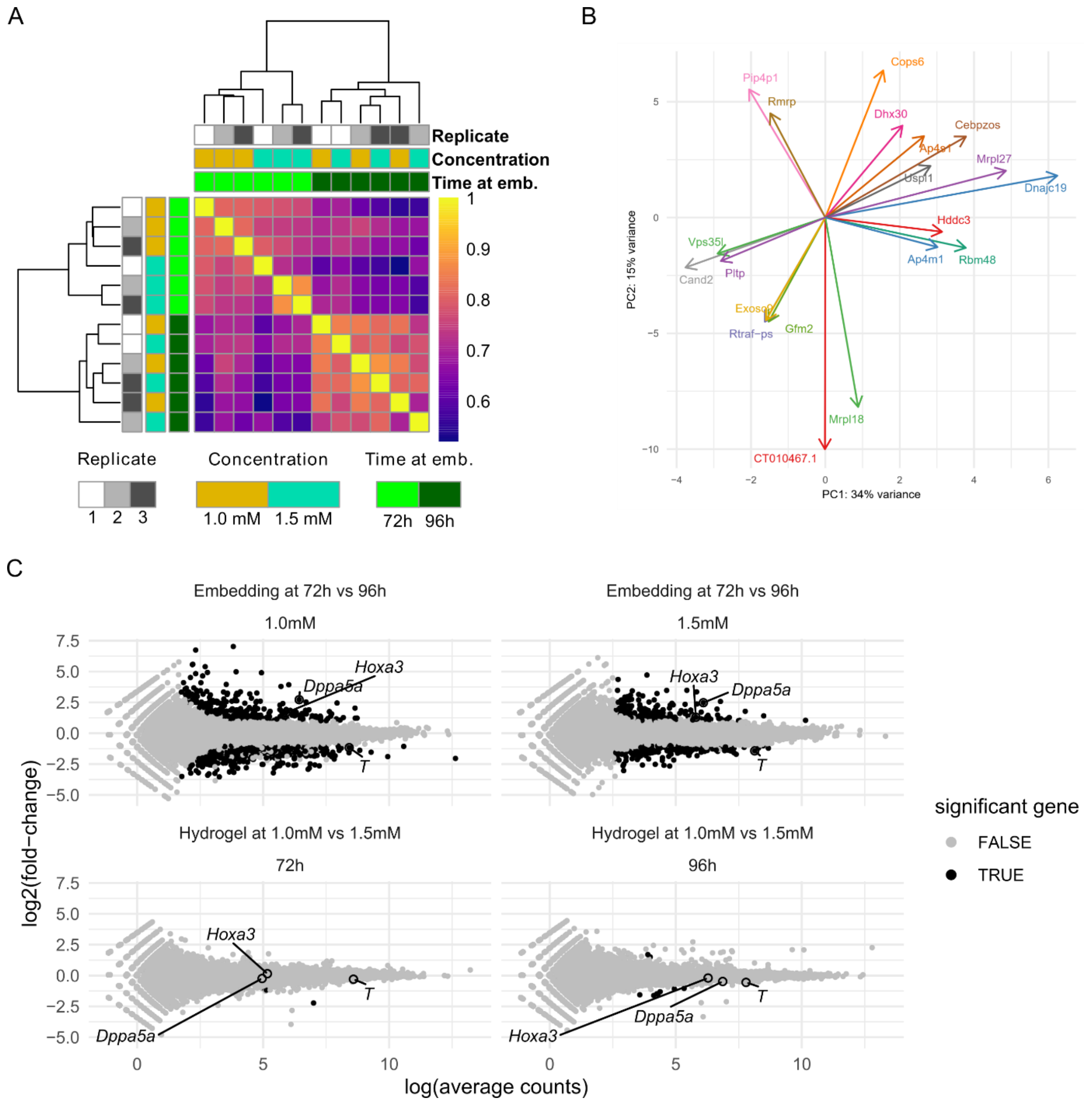

Fig. S9. Detailed RNA-seq analysis on the effect of early embedding and high gel concentration. (A) Correlation matrix of bulk RNA sequencing experiments clustered using the ward-D2 algorithm, using samples embedded at 72 h or 96 h, at gel concentrations of 1.0 mM or 1.5 mM. Colors indicate the Pearson's correlation coefficient values. All samples are analyzed in three independent replicates. (B) Loadings from PCA analysis shown in Figure 3C. (C) MA-plot showing the differential gene expression (log2FC ≥ ± 1, adjusted p-value<0.05 as measure by DESeq2 analysis) determined from bulk RNA sequencing. On the upper row, comparing gastruloids embedded at 72 h vs 96 h, for embedding in 1.0 mM or 1.5 mM gels. On the lower row, comparing gastruloids embedded in 1.0 mM vs 1.5 mM gels, for embedding at 72 h or 96 h.

Development • Supplementary information

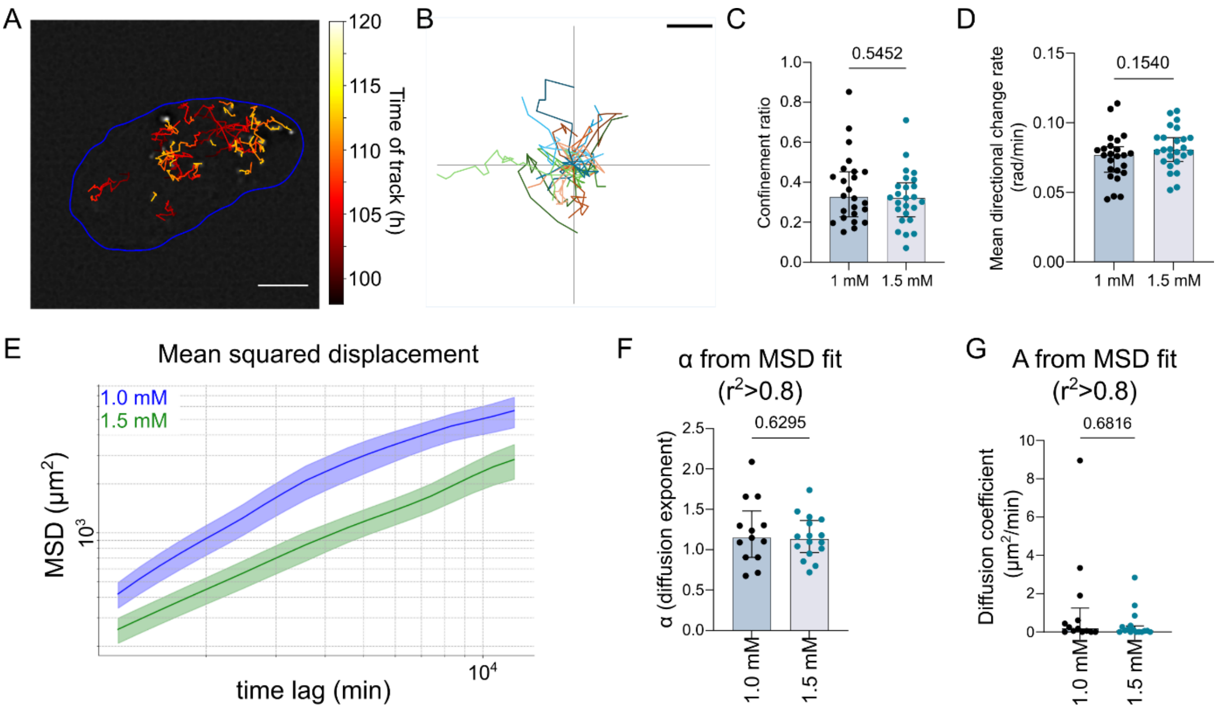

Fig. S10. Analysis of single cell tracking in gastruloids. (A) Tracks of cells in a gastruloid embedded in a 0.8 mM gel, filmed from 98 h to 120 h using a classical epifluorescence microscope. Contour in blue was determined using thresholded brightfield image. Tracks were obtained using trackmate. Colors correspond to the time point at which the tracking segment was found. Scale bar 50 µm. (B) Example trajectories of cells tracked in gastruloids embedded in a 1.0 mM gel from 72 h to 95 h. Gastruloids made from a mix of cells expressing either a reporter of BRA expression (TProm-mVenus cells) alone, or together with a nuclear marker (H2B-iRFP). Tracked cells all expressed H2B-iRFP, without taking into account the TProm-mVenus signal. Trajectories from 2 gastruloids have been pooled and aligned to start at the origin (0,0), so as to show the extent of 2D exploration by cells. Each color represents one trajectory by one cells. Imaging was done using using the LS2 Viventis system. Scale bar 5 µm. (C) Confinement ratio and (D) Mean directional change rate for trajectories analyzed in Figure 4D. (E) Mean squared displacement (Mean ±SEM) of trajectories analyzed in Figure 4D. (F) Diffusion exponent and (G) Diffusion coefficient extracted from fit with r2 > 0.8 of the MSD in Figure S9E. Experiments performed in 1 replicate, with 2 gastruloids per condition. Statistics: Mann-Whitney test.

Development • Supplementary information

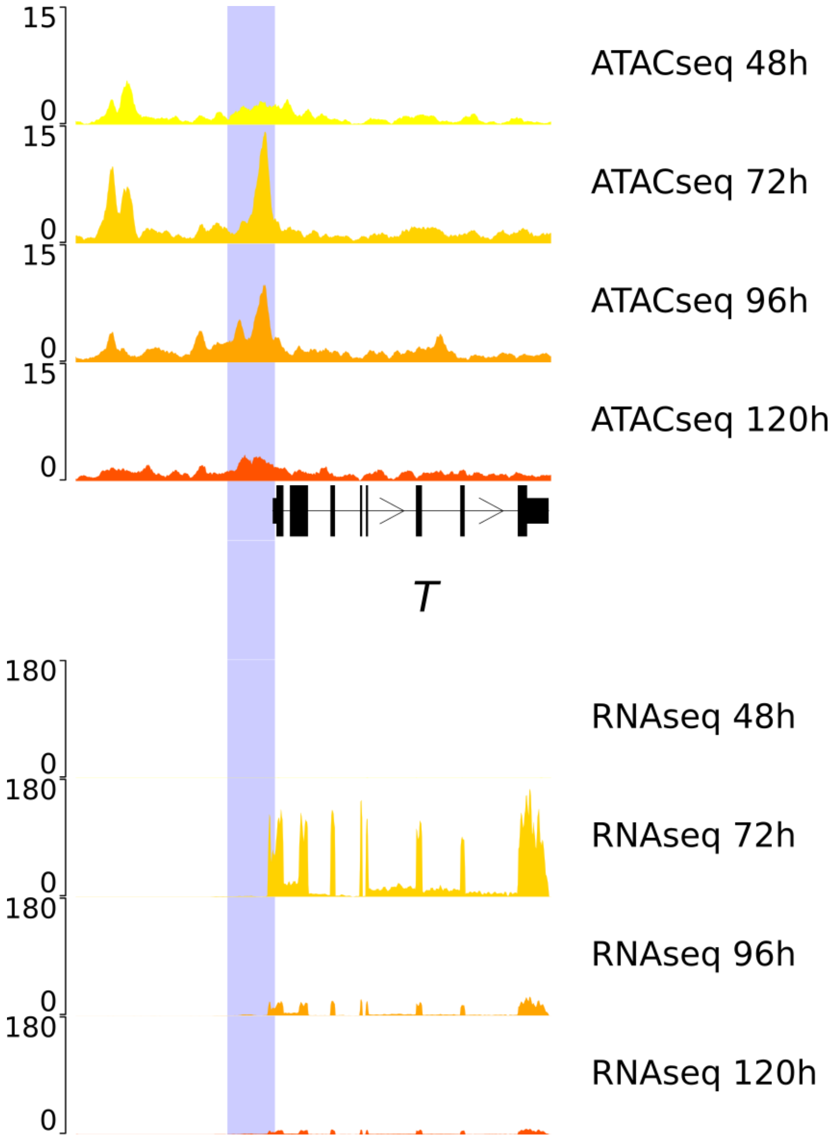

Fig. S11. Genome browser view of ATAC-seq and RNA-seq for generation of the TProm-mVenus cell line. A region of 1392bp surrounding the brachyury was selected to monitor the activity of the brachyury promoter

Table S1. List of differentially expressed genes in the compared conditions the non-embedded condition was used as reference.

Available for download at
https://journals.biologists.com/dev/article-lookup/doi/10.1242/dev.204711#supplementary-data

Table S2. List of differentially expressed genes in all pairwise comparisons relative to the time of embedding or to the gel concentration.

Available for download at
https://journals.biologists.com/dev/article-lookup/doi/10.1242/dev.204711#supplementary-data

Development • Supplementary information

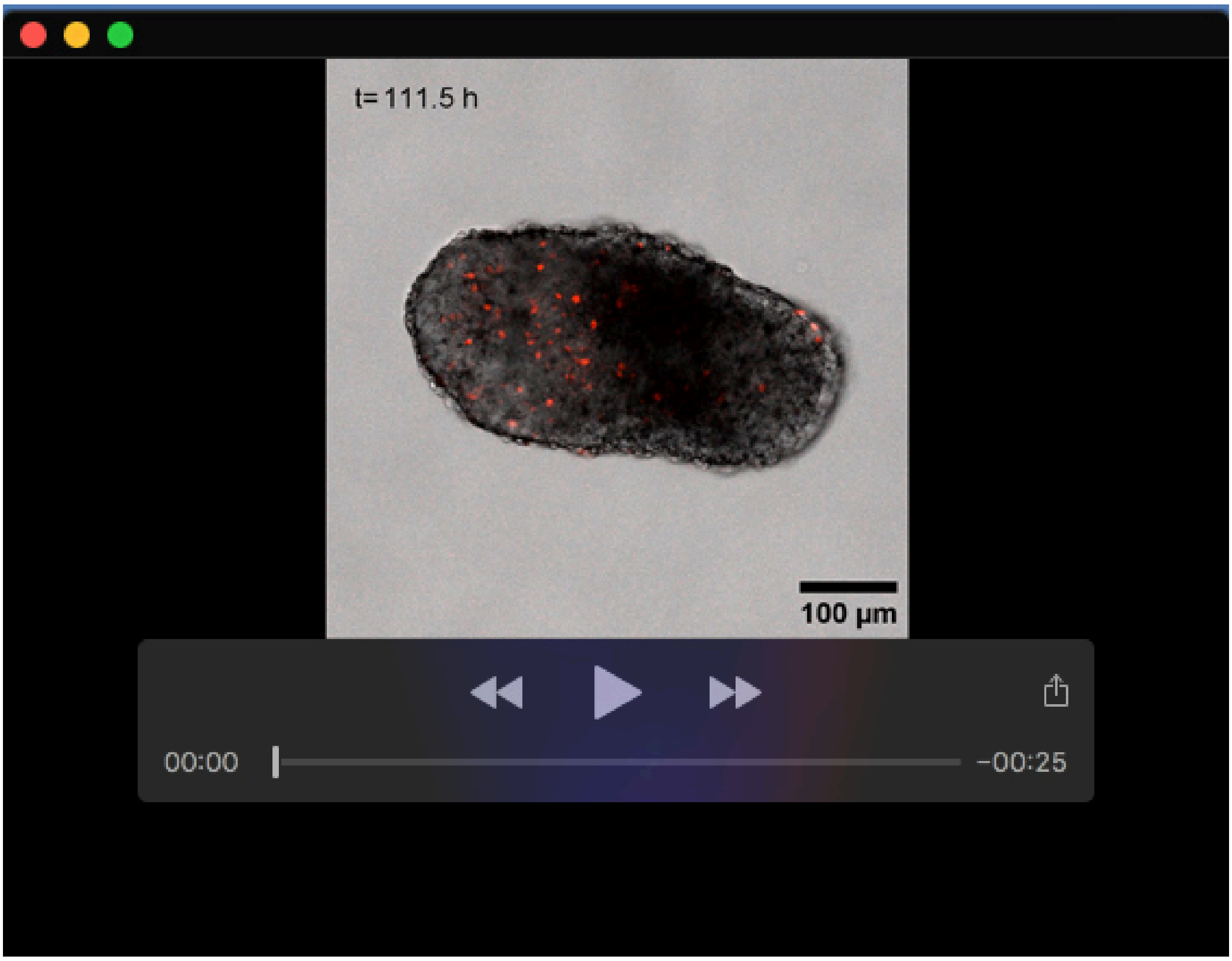

Movie 1. Example of elongating chimeric gastruloid in 0.8mM dextran gel. Chimeras are constituted of 129/svev cells and a small fraction of cells expressing a nuclear marker H2B-iRFP. Video shows bright field intensity with superimposed fluorescence.

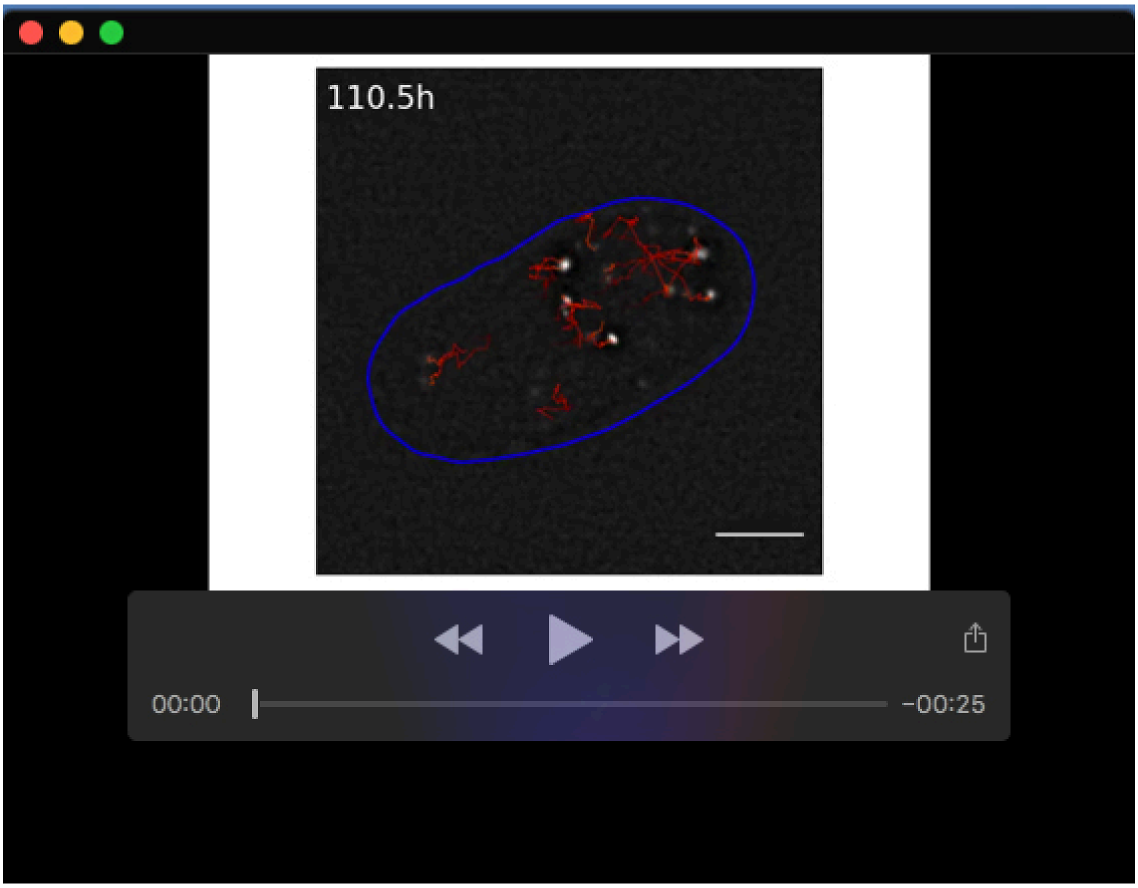

Movie 2. Example of elongating chimeric gastruloid in 0.8mM dextran gel. Chimeras are constituted of 129/svev cells and a small fraction of cells expressing a nuclear marker H2B-iRFP. Gastruloid contour was extracted using brightfield images and plotted in blue. Video shows fluorescence as well as tracks extracted using TrackMate. The same gastruloid was used to produce Figure S10A.

Supplementary Materials and Methods

Sequence used in transgenic assay

ACCCCAGAGGTTGGCTCCTGGAAAACCGTCTCTCCCAGAAGTAGGG
GCAGGTAGAACCCACAACTCCGACCCCAAAGACTTCCCAGGGAGACTC
TCAGAGAGACAACGAACTCAGAATTGAGTGCCCCCACCTGATTCAGGG
GCCTCTTCCAAGGAGCTTCGGGATAGGATAGGAGAGTGGAAGACGGGG
AAACGGAGGCTGGAACCCAGAGTCTCGTTAAAGAGCTGGGCGCGAGCT
CTGGCTTCCTTCCCGCTTCCTGGGCTCCCGTTTTAGAGGAATGTTATTG
TTTAAAGAGACCCCATTGAACTATTTCCTGCTCTTTGTCACCTTCCCCT
CACTCTCCCGGCAGAGGTTCTCACCGAGAGGCAATAAACCAACTGCTG
CCCACACCGCATGGCGAGGCGGGTAGGGAAACGCGCGCAGCATGCGTT
CCAACAATCCCCGGCGCAAAGAGACCAGGGACTCCCGGGGCCACATTC
GGTGCAGGCGCATCCACCGTCAAAGTCCAGCTTTTATGTGGGACGCGA
GGACACCTCCTACTAGGGTCGCTATCTGTTCGTCTATTTCCCTCTCTGG
ACAGATCCGCATTGAGCTTCCCTCTCCACGCAGGTGAAGGTCGTGGGG
GACCTGGATGCCGAGGTGGGAGTTAGTGGCAGTCCATGGGGCGAGGG
GACGTGTCCCAAAGCTGCCACACCTGGGGAGGCTGAGGCTTTGGAGAG
GTCAAGGAGACCCGGGAGACGCCGATCCGCCGAAGTCCCTCTCAGGTG
CGCGCAGCGTGGACACTCCGCGGGGCAAAGTCGCAGGCGCCGGTGTGC
GCTTGGACAGCGCGTGGGAGTGGAGAGTTTAGCAGTGGCTCTAGGAGC
CAGGGTCCTGGGTGGCTCCAGCCCGGCTTCTCGCCCTCCCTCCCCCAG
GGTCCGCCCCGCCGCTTTGATGGAGGTGCAAACATTTGGGGGAGGGCG
GGGGTGTCGGGACTGCGCCCGACGCTTTCCTTACAGGAAGCGCGCGCT
GGAGCCCATTGTTGGCCCCCAGCCTCCGGGCCCGCCCGGCCAGTCTGA
TATGGCCGCGCACCGCCAATGGGCAGCTGCTCGGTACTTCAAAGGGTG
TCCCGCCCAATCCGCCGCACCCCCCTGCGAGGCCACCTCGGCTGTATTT
ATGGGGAGGGGACCCATTTTTCTCTTCCCCAGAGACTTACTCTTGTCG
CGCCTTGCGGGAGTTCAAGTGGAGCCACGGCTCCCCAGGCCCTCTCCC
CCATCCCCGCCCCCTTCCCCCCTCATCCCGATCTCGGTGCTCCTTTGGC
GAATGTGCAGGGACCCAGGTGTAATCTTTGGGCTCCGCAGAGTGACCC
TTTTTCTTGGAAAAGCGGTGGCGAGAGAAGTGAAGGTGGCTGTTGG
